# Listeria monocytogenes (Lm)-LLO immunotherapies reduce the immunosuppressive activity of myeloid-derived suppressor cells and regulatory T cells in the tumor microenvironment

**DOI:** 10.1186/2051-1426-1-S1-O18

**Published:** 2013-11-07

**Authors:** Anu Wallecha, Inga Malinina, Poonam Molli

**Affiliations:** 1 Research and Development, Advaxis Inc., Princeton, NJ, USA

## 

Myeloid-derived suppressor cells (MDSC) and regulatory T cells (Treg) are major components of the immune suppressive cells that potentially limit the effectiveness of an immunotherapy-based treatment. Both of these suppressive cell types have been shown to expand in tumor models and promote T-cell dysfunction that in turn favors tumor progression. In preclinical studies using transplantable mouse models, we observed that live attenuated bioengineered Listeria monocytogenes (Lm)-LLO immunotherapies have an impact on the suppressive ability of MDSC and Treg in the tumor microenvironment (TME), resulting in a loss in the ability of these cells to suppress T cells. This alteration of immunosuppression in the TME was an inherent property of all Lm-LLO immunotherapies tested and was independent of the tumor model. The virtually total loss in the suppressive ability of these cells in the TME was linked to a decrease in the expression of arginase I in MDSC and IL-10 in Treg. We are further investigating if the MDSC are differentiated into functional macrophages that increase antigen presentation within the TME in order to stimulate T cell immunity. Overall, this study provides insight into a potentially novel mechanism of action of Lm-LLO immunotherapies that may contribute to therapeutic anti-tumor responses.

